# Availability of essential diagnostics in ten low-income and middle-income countries: results from national health facility surveys

**DOI:** 10.1016/S2214-109X(21)00442-3

**Published:** 2021-10-06

**Authors:** Harika Yadav, Devanshi Shah, Shahin Sayed, Susan Horton, Lee F Schroeder

**Affiliations:** a Internal Medicine, University of Tennessee College of Medicine, Chattanooga, TN, USA; b Dalla Lana School of Public Health, University of Toronto, Toronto, ON, Canada; c Department of Pathology, Aga Khan University Hospital Nairobi, Nairobi, Kenya; d School of Public Health and Health Systems, University of Waterloo, Waterloo, ON, Canada; e Department of Pathology, University of Michigan School of Medicine, Ann Arbor, MI, USA

## Abstract

**Background:**

Pathology and laboratory medicine diagnostics and diagnostic imaging are crucial to achieving universal health coverage. We analysed Service Provision Assessments (SPAs) from ten low-income and middle-income countries to benchmark diagnostic availability.

**Methods:**

Diagnostic availabilities were determined for Bangladesh, Haiti, Malawi, Namibia, Nepal, Kenya, Rwanda, Senegal, Tanzania, and Uganda, with multiple timepoints for Haiti, Kenya, Senegal, and Tanzania. A smaller set of diagnostics were included in the analysis for primary care facilities compared with those expected at hospitals, with 16 evaluated in total. Surveys spanned 2004–18, including 8512 surveyed facilities. Country-specific facility types were mapped to basic primary care, advanced primary care, or hospital tiers. We calculated percentages of facilities offering each diagnostic, accounting for facility weights, stratifying by tier, and for some analyses, region. The tier-level estimate of diagnostic availability was defined as the median of all diagnostic-specific availabilities at each tier, and country-level estimates were the median of all diagnostic-specific availabilities of each of the tiers. Associations of country-level diagnostic availability with country income as well as (within-country) region-level availability with region-specific population densities were determined by multivariable linear regression, controlling for appropriate covariates including tier.

**Findings:**

Median availability of diagnostics was 19·1% in basic primary care facilities, 49·2% in advanced primary care facilities, and 68·4% in hospitals. Availability varied considerably between diagnostics, ranging from 1·2% (ultrasound) to 76·7% (malaria) in primary care (basic and advanced) and from 6·1% (CT scan) to 91·6% (malaria) in hospitals. Availability also varied between countries, from 14·9% (Bangladesh) to 89·6% (Namibia). Availability correlated positively with log(income) at both primary care tiers but not the hospital tier, and positively with region-specific population density at the basic primary care tier only.

**Interpretation:**

Major gaps in diagnostic availability exist in many low-income and middle-income countries, particularly in primary care facilities. These results can serve as a benchmark to gauge progress towards implementing guidelines such as the WHO Essential Diagnostics List and Priority Medical Devices initiatives.

**Funding:**

Bill & Melinda Gates Foundation.

## Introduction

To benefit from essential medicines, a patient must have an accurate diagnosis. Without access to diagnostics, including pathology and laboratory medicine (PALM) diagnostics and diagnostic imaging, patient management relies on syndromic diagnosis and empiric treatment.^1^, ^2^ A recent series of *Lancet* reviews have described the lack of access to diagnostics and the roadmap to improvement.^3^, ^4^, ^5^

While there has been a significant improvement in diagnostic support for HIV/AIDS, tuberculosis, and malaria in low-income and middle-income countries (LMICs) over the past decades, significant gaps persist in availability and quality, even for diseases of public health priority.^6^, ^7^, ^8^, ^9^

Although several studies have documented diagnostic availability within different countries and settings,^10^, ^11^, ^12^, ^13^ few have used a common methodology to analyse diagnostic availability between countries. One such study^14^ extracted data from the Survey Provision Assessment (SPA) in the Demographic and Health Surveys (DHS) in ten countries and assessed 50 items that the WHO considers essential for providing health care, including eight diagnostic tests. The authors found only 2% of facilities provided all these essential diagnostics.^14^ However, a limitation of this study was that only eight laboratory diagnostic tests were included (with no diagnostic imaging) and that analysis did not stratify by specific test.

We aimed to analyse SPA surveys to comprehensively detail essential diagnostic capacity. We assessed availability by health facility tier, by region within-country, and by associating availability to country incomes, population densities, and trends over time for 16 PALM and imaging diagnostics. These findings can inform ongoing policy efforts to improve diagnostic services in LMICs.

**Research in context**

**Evidence before this study**

There has been no previous study focusing on general diagnostic availability across multiple countries using a nationally representative survey tool. The Service Provision Assessment is a health facility survey providing an overview of a country's health service readiness and includes questions on laboratory and radiological diagnostic capacity. One report summarising SPA findings across all components of health care included a brief assessment of laboratory capacity, finding that only 2% of facilities across ten countries adequately offered all eight tests selected for analysis in the study, but it did not stratify availability by test and did not include radiological examinations. With the establishment of the WHO Essential Diagnostics List in 2018 and growing international consensus that improved laboratory and radiological investment is crucial, there is a need to establish a baseline against which future investments in diagnostic capacity can be measured.

**Added value of this study**

This study provides an in-depth evaluation of the availability for 16 laboratory and radiological diagnostics across ten low-income and middle-income countries. We found diagnostic availability in general to be limited, but that it varied greatly by the type of diagnostic, the tier of the facility within the health system, and country. We also found that in primary care, but not hospitals, availability tended to be lower in countries with lower per capita income and in regions with lower population density within each country.

**Implications of all the available evidence**

The study finds the overall availability of essential diagnostics to be lacking at the time of the surveys in many low-income and middle-income countries, but that there is relatively higher capacity that exists for some diagnostics, particularly at the hospital level. Therefore, policies should aim to improve diagnostic capacity in general but also to ensure a focus on primary care. This analysis can act as a baseline against which the impact of future investments in diagnostic services can be measured.

## Methods

### Study design

In this study, we extracted health facility data from the SPA database of USAID, which provides an evaluation of health services capacity in countries, including clinical laboratory testing and diagnostic imaging. The number of facilities in SPA surveys is powered to be representative of facility type and within-country region (eg, site visits to 10% of all facilities in the country, with weighting factors provided for national estimation), although in some cases all facilities are surveyed (Haiti, Malawi, Namibia, and Rwanda). Facility types describe the level of care (eg, from basic primary care facilities such as health posts and pharmacies to tertiary care hospitals). Regions typically represent level one administrative political boundaries. SPA surveys include facilities of public and non-public ownership and are cross-sectional. This study used publicly available data and was not subject to approval by an ethics committee.

### Procedures

Questions about diagnostics in SPA surveys typically included the following: “Is the test reported to be done at the site?”; “is all the equipment used for the test available and observed by a surveyor?”; and “is all the testing equipment in working condition?”. In this study, a diagnostic was considered available at the facility if the answer to each question was affirmative for all three questions, or if records of specimen transport of samples to higher tiers were available. A set of 16 diagnostics (including all three available imaging modalities [eg, x-ray, ultrasound, and CT]) were included in this study, chosen by expert opinion of the coauthors, while each PALM diagnostic is on the WHO Essential Diagnostics List v3 (EDL).^15^ The EDL recommends different tests for different levels of capacity in a health system. We therefore chose different sets of diagnostics to be expected at different tiers. This assignment was informed by the EDL, which lists tests for facilities with and without laboratories, as well as the WHO recommendations for a Positive Pregnancy Experience, which lists antenatal care diagnostics in primary care.^16^ Therefore, at primary care, we assigned diagnostics for HIV, malaria, urine glucose and protein, urine pregnancy, syphilis, microscopy, haemoglobin, and glucose by glucometer, as well as ultrasound. At the hospital level, we additionally assigned Gram stain, chemistry analysers, haematology analysers, tuberculosis, x-ray, and CT (appendix p 4). Glucometers and haemoglobin were removed from the hospital level list because these tests are included on chemistry and haematology analysers.

At the time of data extraction (February, 2020), DHS SPA surveys were available for ten countries: Bangladesh, Haiti, Kenya, Malawi, Namibia, Nepal, Rwanda, Senegal, Tanzania, and Uganda. Abbreviated surveys (eg, HIV SPA) were excluded except for the Maternal and Child Health and HIV SPA performed in Kenya in 2004, which allowed an earlier timepoint comparison. Overall, two surveys from different timepoints were included for four of these countries: Haiti, Kenya, Senegal, and Tanzania. Surveys spanned 2004–18 and included 8512 facilities across all surveys. These countries represent a range of LMIC economies of varying geographical size and development status. SPA surveys are typically recoded to facilitate comparison by year and country and there have been several versions of recoding over the years. Minor adjustments in our analysis allowed for comparison between recode versions. The survey in Congo from 2018 was excluded because it was not recoded. We recoded the Senegal 2017 survey to facilitate comparison to the Senegal 2012 survey.

Each SPA survey publishes a report and includes estimates of diagnostic availability. Countries might choose to calculate availability in different ways. For example, one country might choose to calculate availability of tuberculosis sputum testing as a percentage of all health facilities, while another might calculate as a percentage of all health facilities that offer tuberculosis services. To facilitate comparison between countries, we recalculated each diagnostic's availability using the same criteria for every country (appendix p 4), even if these calculations did not match each individual country's published report. When data were missing for a given question (eg, “NA” in data fields), we assumed the test was not available as the relevant service was not offered, consistent with a previous study of these same data (where 36% of diagnostic data fields were “NA”).^14^ Facilities with zero weighting were excluded (210 of 8512 facilities; no explanations were provided in SPA datasets). When considering availability of a diagnostic, any test format was accepted including specimen transport (provided logs were observed). For example, tuberculosis testing would be satisfied by on-site acid-fast bacteria sputum microscopy or nucleic acid testing (or specimen transport for either).

Each country used similar but unique health system tiers. To compare results between countries, country-specific tiers were mapped onto one of three generalised tiers: basic primary care, advanced primary care, or hospital (see appendix p 5 for tier assignments). Where facility descriptions were available, primary care facilities with trained staff but no medical doctors or nurses were considered to be in the basic primary care tier, and those with doctors or nurses were considered to be in the advanced primary care tier. As an example, in Tanzania, health posts and dispensaries were merged into the basic primary care tier, health centres represented the advanced primary care tier, while all hospital types were merged into the hospital tier. For Senegal, we excluded Case de Santé facilities as they are an extension of basic primary care into communities with care delivered by community health workers, and the SPA evaluation of diagnostics for these facilities was limited to malaria.

### Statistical analysis

Percentages of facilities offering each diagnostic test were calculated from SPA data, accounting for facility weights, stratifying by tier, and for some analyses, region. The tier-level estimate of diagnostic availability was defined as the median of all diagnostic-specific (eg, haemoglobin or ultrasound) availabilities at each tier, and country-level estimates were the median of all diagnostic-specific availabilities at each of the tiers. All references to changes in availability refer to absolute changes, such that a 5 percentage points increase from 50% would be 55%. Some diagnostics were not in some surveys and were excluded from the calculations (eg, glucose meters in Kenya and HIV testing in Bangladesh). Where multiple laboratories were present in a single facility, diagnostic capacity in any one laboratory was sufficient to consider the diagnostic available. Median diagnostic availability in different within-country regions were evaluated with interquartile ranges (IQR), quartile coefficients of dispersion, and outlier detection. Outliers were defined with availabilities beyond 3 median absolute deviations (MAD; the median of the absolute value of each point's deviation from the median) from the within-country median.^17^ MAD was calculated using the mad() command in R using a scaling factor of 1·4826 so that, for normally distributed data, MAD equals standard deviation. Associations of country-level diagnostic availability with country income (World Bank gross-domestic product per capita at constant prices of 2019) and (within-country) region-level availability with region-specific population densities were determined by multivariable linear regression, controlling for appropriate covariates including tier. To avoid extreme values and to facilitate comparison between countries, incomes were log10-transformed and region-specific population densities were centred and scaled within-country. Change of availability associated with a 10% change in income was calculated as log10(1 + 0·1) × (regression coefficient). Only the most recent survey for each country was included for all analyses other than comparisons of availability over successive surveys within the same country. All analyses were performed in R statistical environment (v.3.6.3).

### Role of the funding source

The funder of the study had no role in study design, data collection, data analysis, data interpretation, or writing of the report.

## Results

**Figure 1:**
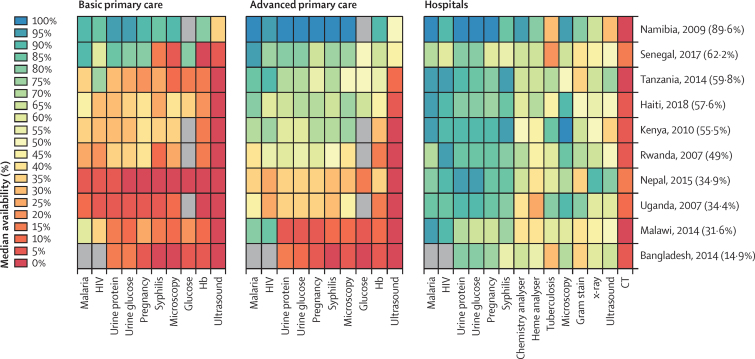
Availability of diagnostics by tier and country The heat map provides information on the proportion of facilities offering the diagnostic. Percentages in parentheses after each country name is the median diagnostic availability of all cells in each row. For countries with more than one survey, only the most recent was included. Countries are ranked in descending order of median availability. Availability are also sorted left to right in decreasing order of availability across countries.

Diagnostic availability varied considerably by diagnostic and by tier (figure 1). Median availability of diagnostics expected to be found at the respective tiers increased at higher tiers in the health system: basic primary care (19·1%, IQR 6·4–36·7), advanced primary care (49·2%, 18·1–75·0), and hospital (68·4%, 51·1–84·6). The most available diagnostics, regardless of tier, included those for HIV, malaria, urine protein, urine glucose, urine pregnancy, and, at the advanced primary care and hospital tiers, those for microscopy and syphilis (appendix p 8). Tests available in instrument-free point-of-care formats (eg, malaria, HIV, urine protein and glucose, pregnancy, and syphilis) showed more availability whereas diagnostic imaging was among the least available within each tier. Availability based on specimen transport was minimal and only for some diagnostics: tuberculosis (6·6% median availability between all country-tier availabilities), HIV PCR (3·5%), HIV ELISA (2·7%), basic chemistries (1·6%), Gram stain (1·1%), urinalysis (1·0%), malaria (0·8%), HIV western blot (0·7%), and HIV rapid (0·3%).

Across tiers, Namibia had the highest country-level availability of PALM and imaging diagnostics (89·6%), followed by Senegal (62·2%), Tanzania (59·8%), Haiti (57·6%), Kenya (55·5%), Rwanda (49·0%), Nepal (34·9%), Uganda (34·4%), Malawi (31·6%), and Bangladesh (14·9%; figure 1). However, between-country rankings of tier-level availability varied by tier, with Senegal ranking second highest in availability in both basic and advanced primary tiers but lowest in availability at the hospital tier. By contrast, Nepal ranked lowest in basic primary and third lowest in advanced primary, while ranking third highest in the hospital tier (appendix p 9). Country-level availabilities were correlated (spearman's rank correlation coefficient) between basic and advanced primary care tiers (ρ=0·92), but not between other tier-pairs (appendix p 2).

**Figure 2:**
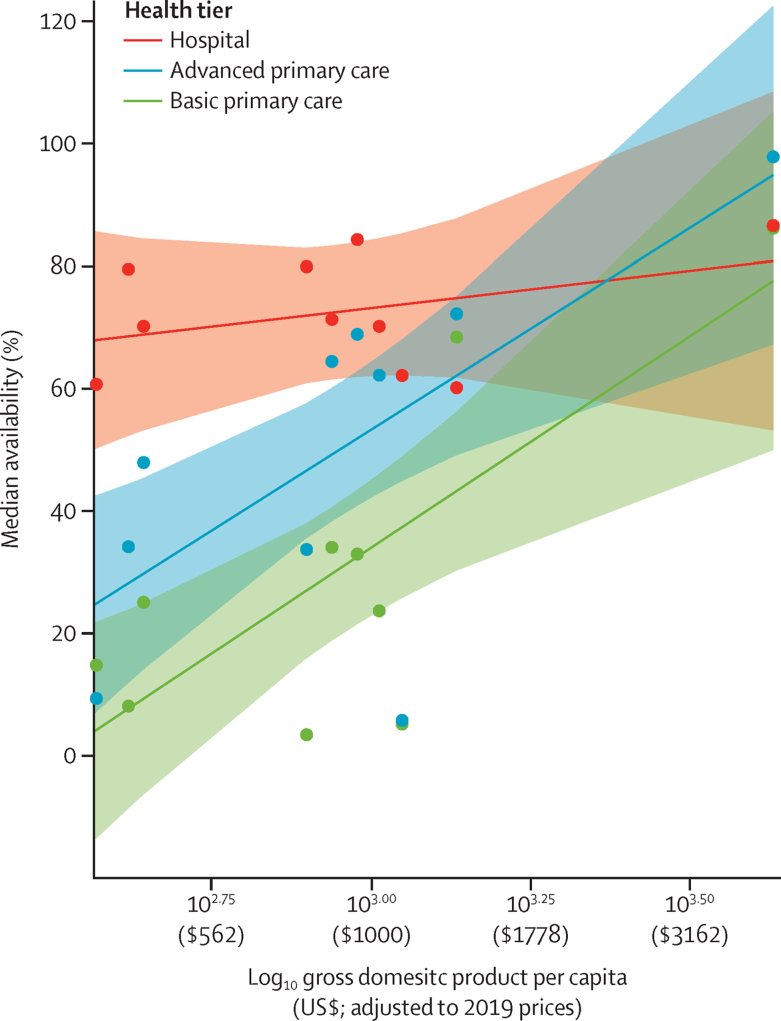
Availability by GDP per capita Plot of marginal effects showing the association between median country-level diagnostic availability by tier with income, after adjusting with a covariate for year of survey. The interaction of income and tier had p values of 0·044 for basic primary care and 0·057 for advanced primary care tiers (appendix p 10). The interaction between tier and income is evidenced by more sloped lines at primary care tiers as compared with the hospital tier. Separate linear regressions performed independently for each tier, with Year as covariate, produced significant coefficients for basic primary care (p=0·00155), advanced primary care (p=0·0345), but not hospital (p=0·1996) tiers. GDP=gross domestic product.

Multivariable linear regression of median availability against log10(income), tier, year of survey, and the interaction of log10(income) and tier, showed a positive effect of log10(income) at both primary care tiers compared with hospital tier (figure 2 and appendix p 10). For each increase in income of 10 percentage points, there was an associated absolute increase of 2·4 percentage points availability at the basic primary care tier and of 2·2 percentage points availability at the advanced primary care tier.

**Figure 3:**
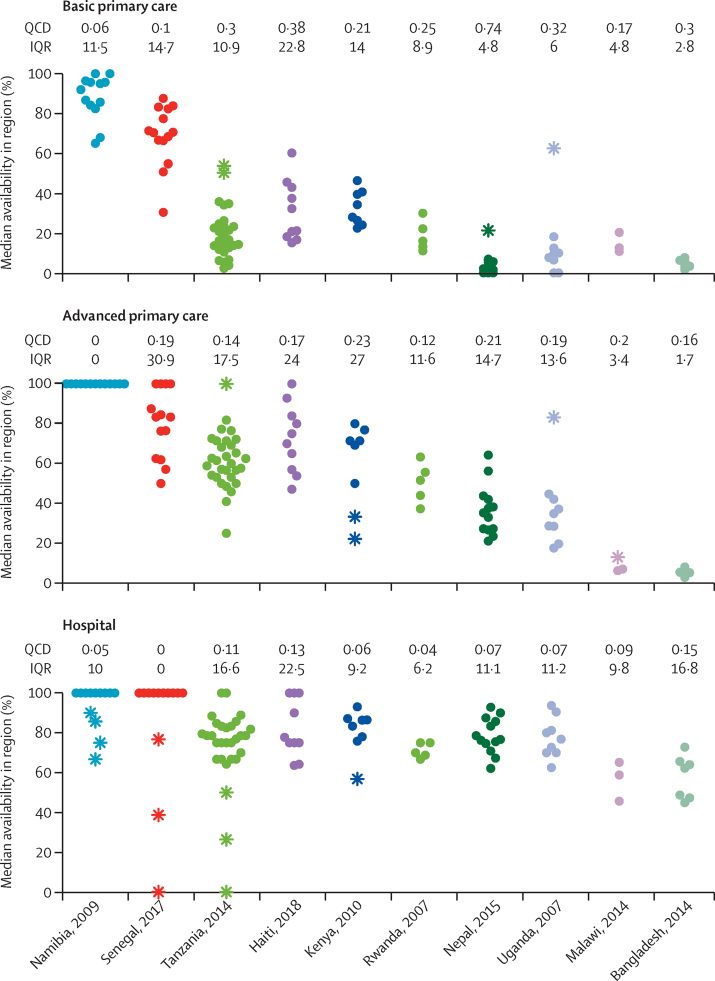
Variation of diagnostic availability among regions of each country, by tiers In this dot plot, the IQR and QCD of region-specific availabilities are calculated for each country and tier. Outliers are depicted with asterisks and determined as described in the methods section. QCD=quartile coefficient of dispersion.

**Figure 4:**
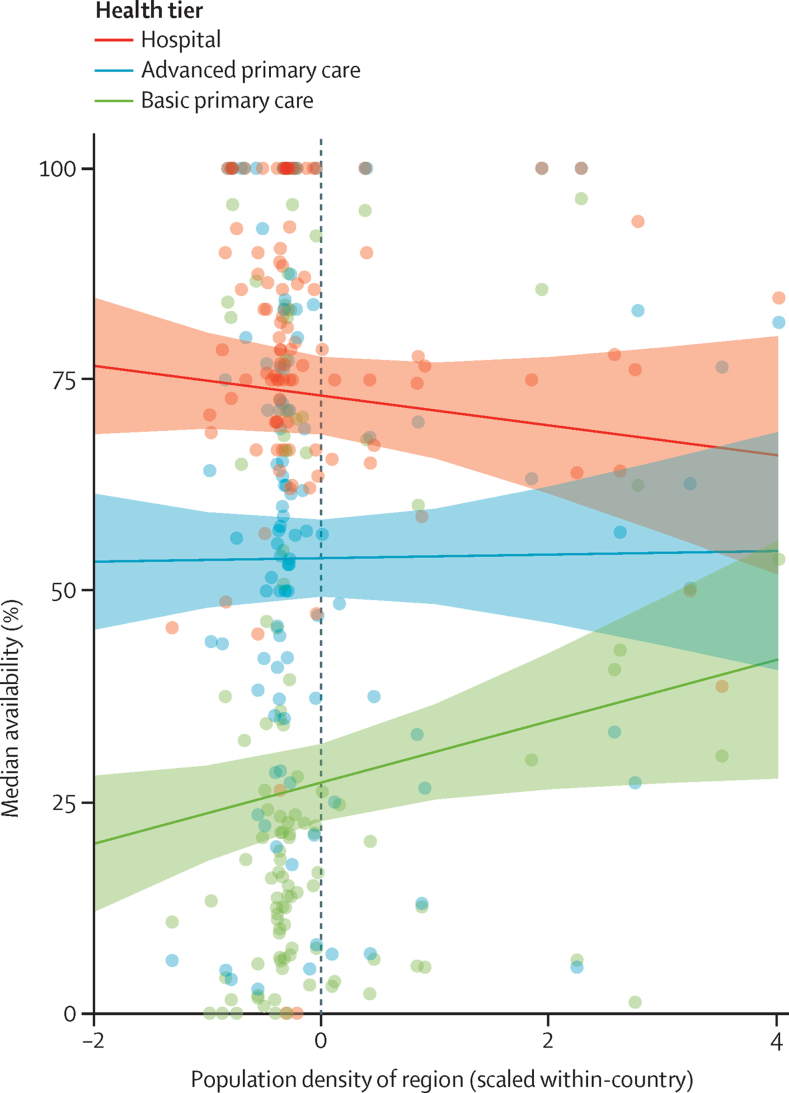
Region-specific availability by regional population density, by tiers Plot of marginal effects showing the association between region-level diagnostic availability and population density by tier. The interaction of population density with tier is shown by a more sloped line at the basic primary care tier than other tiers. Since the regional population density is Z score transformed within-country, the regression coefficient of 5·4 for the interaction term population density:basic primary care means a Z score population density shift of 1 is associated with an increase of 5·4 percentage points in availability at the basic primary care tier (eg, shift from 50·0% to 55·4%). See appendix p 16.

In evaluating equity of access, variation of diagnostic availability within-country was calculated through analysis of region-specific availabilities of diagnostic methods at different tiers (figure 3). Most countries had at least one outlier region when summing up outliers across tiers: Tanzania (six outliers), Namibia (four outliers), Kenya (three outliers), Senegal (three outliers), Uganda (two outliers), Nepal (one outlier), Malawi (one outlier; see appendix p 1). At the basic primary care tier there were four outliers overall (all with greater availability). At the advanced primary care tier there were five outliers overall (three with greater availability). At the hospital tier there were 11 outliers overall (none with greater availability). No outlier regions were found in Haiti, Rwanda, or Bangladesh. IQRs of regional availability within country and tier spanned from 0% to 30·9%. The median IQR for all countries was greatest at the advanced primary care tier (14·2%), and slightly lower at the basic primary care (9·9%) and hospital (10·6%) tiers. Only one country, Haiti, showed both a relatively high IQR and quartile coefficient of dispersion (appendix p 12). Multivariable linear regression including normalised population density, tier, country, and the interaction of tier and population density found region-specific population density to be positively associated with availability of diagnostics at the basic primary care tier as compared with the hospital tier (figure 4, appendix p 12).

Four countries were evaluated for diagnostic availability over time (Haiti, Kenya, Senegal, and Tanzania). There was an increase in country-level diagnostic availability in all countries, ranging from 3·7 to 14·9 percentage points (table; appendix p 3). For each country and each tier, there was always a positive change, except Tanzania and Kenya at the hospital tier, which showed a decrease of 15·1 and 3·5 percentage points, respectively. Otherwise, diagnostic availability increased between 2·8 and 50·4 percentage points across health facility tiers, with greatest improvements seen in the basic primary and advanced primary care tiers (table). Most tests showed an increase in availability at the basic primary care tier: 100% of tests (Haiti), 100% (Kenya), 80% (Senegal), 88% (Tanzania). Similarly, most tests showed an increase in availability at the advanced primary care tier: 90% (Haiti), 100% (Kenya), 80% (Senegal), and 88% (Tanzania). Results were mixed at the hospital tier, with increases for 100% (Haiti), 55% (Kenya), 57% (Senegal), and 25% (Tanzania) of tests.

**Table:** Change in median overall diagnostic availability over time by tier, by country

	**Basic primary care**	**Advanced primary care**	**Hospital**	**Overall**
Haiti (2018/2013)	6·9 pp	10·2 pp	13·9 pp	8·8 pp
Kenya (2010/2004)	5·5 pp	30·8 pp	−3·5 pp	14·9 pp
Senegal (2017/2012)	50·4 pp	7·4 pp	2·8 pp	10·6 pp
Tanzania (2014/2006)	6·0 pp	7·8 pp	−15·1 pp	3·7 pp

pp=percentage point.

## Discussion

This study of ten LMICs has found that most essential diagnostic services in the majority of countries undergoing the Service Provision Assessment were very limited, when comparing with WHO standards such as the Essential Diagnostics List (EDL) and the Positive Pregnancy Experience guidelines.^15^, ^16^ Even for the most available tests, such as HIV testing, median availability was under 40% in the basic primary care tier. At the hospital tier, many essential diagnostic tests had an availability of 46–62%, including basic chemistry tests, automatic complete blood count, Gram stain, tuberculosis testing, x-ray, and ultrasound. This is consistent with other landscaping studies that demonstrate low availability of essential diagnostics as listed in the WHO's Essential Diagnostics List.^10^, ^11^, ^12^, ^13^, ^18^

This pattern of availability is not surprising. Enormous international funding has been devoted to HIV and malaria programmes, which have the most readily available diagnostics at primary health-care level. Other diagnostics found to be most available in this study are available in inexpensive, point-of-care formats such as dipsticks and lateral flow assays. PALM and imaging diagnostics outside of vertical programmes that require investment for equipment as well as staffing were found to have lower availability.

Median availability of diagnostics increased with increasing health facility tier: basic primary care (19·1%), advanced primary care (49·2%), and hospital (68·4%). In many LMICs, primary health care usually comprises health posts and dispensaries that serve a smaller population, are located rurally, are staffed by personnel with limited training, and most often do not have laboratories. These factors all contribute to the lack of diagnostic capacity at lower health system tiers. Furthermore, budget lines in Ministries of Health often do not provide protected funding for diagnostics.^19^ According to the Maputo Declaration of 2008, laboratory networks should be developed such that lower tier facilities can send laboratory specimens to higher tier facilities. Our study gave credit for so-called send-out testing to higher tier facilities but specimen referral was rarely offered. We therefore conclude that laboratory networks were largely not functional at the time of surveys. Although teleradiology does offer the ability for remote reading by radiologists, there is a critical lack of the basic imaging equipment required.

Generally, availability ranking between countries persisted when each tier was analysed separately. However, there were exceptions as Senegal had the second highest availability at the primary tiers but lowest availability at the hospital tier, indicating that more resources were distributed to the primary sector than to higher sectors. This correlates with findings in another study that showed that, in accordance with the national referral policy of Senegal, efforts were made to increase basic health services in the public sector.^20^ Multiple public-private partnerships were developed to extend the range of services that could be offered within the public health system due to the Senegalese National Financing Strategy for Universal Health Coverage. By contrast, Nepal shows the opposite pattern. It had the second highest availability of diagnostics at the hospital tier but lowest availability at the primary levels. This pattern might be a result of the Nepalese health system being driven by multiple donor organisations mainly developing disease-driven vertical programmes, while capacity building with generalised medical equipment has not been prioritised within the government-run primary health sector.^21^ This lack of priority to the primary health-care sector might explain why populations largely circumvent closer facilities favouring higher levels of care even for essential services such as antenatal care and child birth .

Namibia showed the highest availability across all tiers, perhaps because of its high per capita GDP but also because of the investment in diagnostics and establishment of the Namibia Institute of Pathology (NIP), which acts as a reference laboratory system for state health services. The NIP was established as a public enterprise in 2000, taking control of 23 laboratories from the Ministry of Health and Social Services at a time when these facilities were facing staffing and infrastructural challenges.^22^ By contrast, Bangladesh, which has the third highest per capita GDP in this analysis, ranked last or second-to-last in availability in each tier. This low ranking might be due to the fact that domestic general government health expenditure is only 17% of current total health expenditure (and the third lowest among the study countries, according to the World Bank). Nonetheless, when regressing against income, availability of diagnostics across all countries were positively associated with income, although only in primary care. One interpretation of this difference for primary care is that only in countries with greater income do investments in diagnostics continue down from tertiary to primary care. Critically, the WHO has emphasised the importance of improving primary care in achieving universal health coverage,^23^ and that improvement includes appropriate diagnostics.

Based on a relatively large regional IQR and quartile coefficient of dispersion, inequality of diagnostic availability was greatest for Haiti's basic primary care tier, possibly because of inequitable or incomplete reconstruction efforts after hurricane Matthew devastated much health-care infrastructure 2 years before the survey. There were 20 outlier regions identified across all ten countries and tiers (of 334 region-tier pairs in total). Six of seven outliers with higher availability were from regions with population densities above the 75th percentile for the country. By contrast, population density did not appear to predict outlying regions with low availability, as only three such regions had population density below the country-specific 25th percentile (appendix p 11). In Kenya for example, both Nairobi (capital city) and northeastern regions were outliers with low availability of diagnostics at the advanced primary care level. In urban centres like Nairobi, patients might prefer hospitals rather than primary health centres because they know there are more comprehensive services available at the hospital. Most of the diagnostics in Kenyan urban cities such as Nairobi are offered in a vibrant private sector both in hospitals and in stand-alone PALM and diagnostic imaging centres while investment in the public health-care sector, especially in diagnostics, is not prioritised. Furthermore, as part of Kenya's Vision 2030, the plan to improve health care was to systematically reduce the government's role in service provision while encouraging private sector investments. By contrast, the northeastern region of Kenya is sparely populated and historically marginalised^24^ thus leading to a regional imbalance in economic, social, and political development. Multivariable regression found region-specific availability to be positively associated with population density, but only in the basic primary care tier. This difference might again be due to diagnostics investment occurring first in higher tier facilities and only last in primary care, with this effect being amplified in low-population regions likely to be less wealthy and more remote.

As for trends over time within country, our study found that each of the four countries with multiple timepoints indeed modestly improved availability from one survey to the next, consistent with real increases in per capita GDP for each country between surveys. While Haiti's per capita GDP growth was small, improvement might have been due to external assistance in the rebuilding effort after the 2016 hurricane. The greatest increases were in basic primary care (ranging from 5·5 to 50·4 percentage points) and advanced primary care (from 7·4 to 30·8 percentage points) compared with hospitals (from −15·1 to 13·9 percentage points). Notably, Senegal basic primary care availability increased dramatically by 50·4 percentage points, consistent with health investments discussed above, though the mean of diagnostic-specific availabilities only increased by 17·6 percentage points.

There were several limitations to this study. This study only evaluated availability in facilities, not necessarily accessibility for a population. If a patient does not have access to facilities, then they would not have access to the diagnostics. As an example, Namibia has poor accessibility to facilities (in terms of access within 2 h for the population),^25^ but high availability of diagnostics at the facilities. This study also did not evaluate the number of tests performed or whether skilled staff were available, only that there was functioning equipment. It is possible that due to affordability or preferences from the provider or patient, these diagnostic services were not appropriately utilised. Also, SPA surveys are not typically conducted with high frequency, thus we relied on data ranging from 2004 to 2018. Importantly, there have been successful diagnostics capacity initiatives after surveys. As an example, Uganda has implemented a hub-and-spoke referral system to the Uganda National Public Health Laboratory Service (UNPHLS), which provides country-wide testing for multiple conditions, including HIV, tuberculosis, and epidemic-prone diseases, as well as haematology and chemistry tests.^26^ The effect of that network was not captured in the 2007 SPA survey. However, the SPA survey can be used to benchmark the improvement of the UNPHLS initiative if an additional survey were to be conducted. Such studies should occur as they do with respect to medicines. Medicines from the WHO Essential Medicines List are routinely surveyed for availability and cost globally, as per resolution of WHO Member States at the 54th World Health Assembly in 2001, calling for the development of “systems for voluntary monitoring drug prices and reporting global drug prices” (Resolution WHA 54·11).^27^

The DHS Service Provision Assessments allow a unique opportunity to landscape diagnostic availability across multiple LMICs, using a representative survey conducted similarly in each country. When benchmarking to the WHO Essential Diagnostics List and the WHO Positive Pregnancy Experience guidelines, this study has shown there remain large gaps in availability at each tier in health systems, with particular deficits in primary care. However, we hope this study will help establish a baseline against which progress in diagnostics delivery can be measured as countries adopt national-level essential diagnostics lists. Successful delivery of universal health coverage must include essential diagnostic availability throughout health-care systems. This availability must be accompanied by accessibility for patients to these diagnostics, as well as high quality and timely results. These efforts will require financing, strengthening of infrastructure, human resource development, and national policies to establish evidence-based use of diagnostics to ensure the greatest impact on patient care.

## Data sharing

Raw data from this study are available at no cost from The DHS Program of USAID.

## Declaration of interests

We declare no competing interests.
