# Supplementary material for: Availability of essential diagnostics in ten low-income and middle-income countries: results from national health facility surveys
Source: Lancet Glob Health. 2021 Oct 6;9(11):e1553–60. doi: 10.1016/S2214-109X(21)00442-3 (PMC8526361; doi:10.1016/S2214-109X(21)00442-3)
Supplement: Supplementary appendix [file mmc1.pdf]

THE LANCET
Global Health

Supplementary appendix

This appendix formed part of the original submission and has been peer reviewed. We post it as supplied by the authors.

Supplement to: Yadav H, Shah D, Sayed S, Horton S, Schroeder LF. Availability of essential diagnostics in ten low-income and middle-income countries: results from national health facility surveys. *Lancet Glob Health* 2021; published online Oct 6. http://dx.doi.org/10.1016/S2214-109X(21)00442-3.

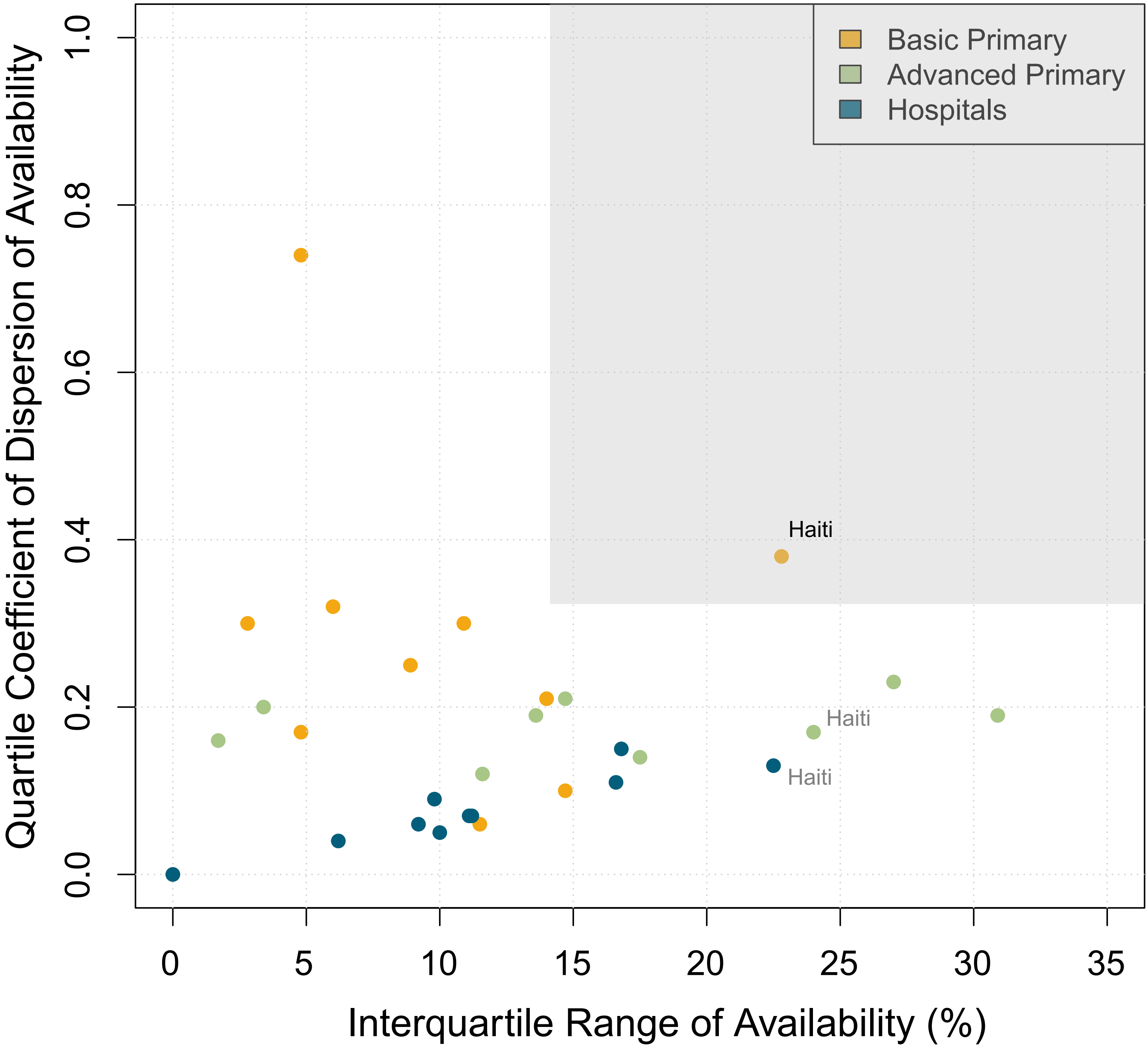

Figure S1. Variation of diagnostic availability between regions within countries. Each dot represents a health system tier of a single country, plotted by the interquartile range (IQR) and quartile coefficient of dispersion (QCD) of diagnostic availability of that country and tier. Shaded region identifies interquartile range > 14.1% and QCD > 0.33, equivalent to the 80th percentile of each measure.

Figure S2a. Paired scatterplots of country-level median availabilty for each health system tier.

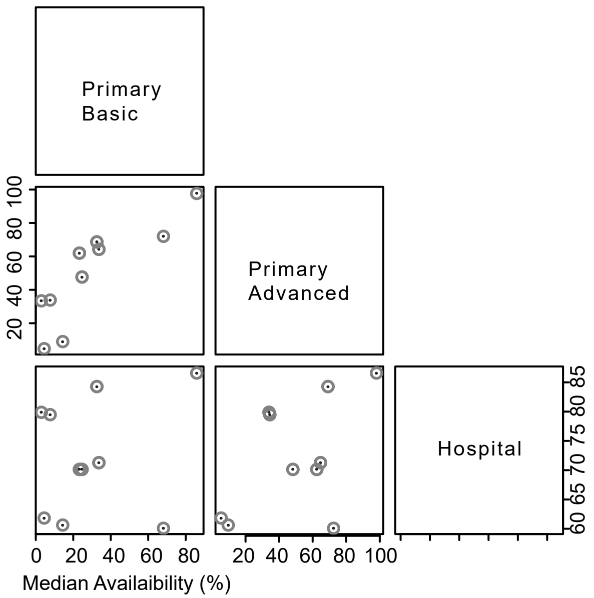

Figure S2b. Spearman rank correlation coefficients comparing country-level availability by health system tier. An 'X' in a tile represents spearman rank correlation coefficients associated with p-values > 0.05.

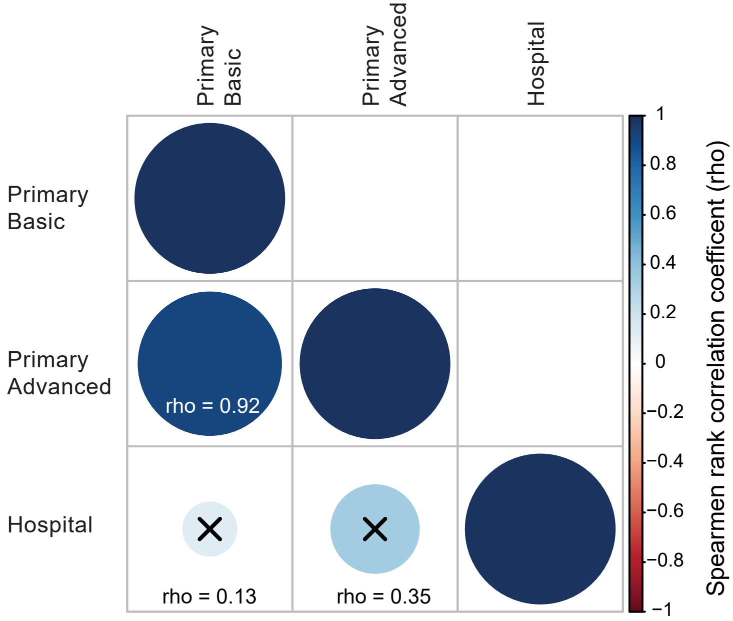

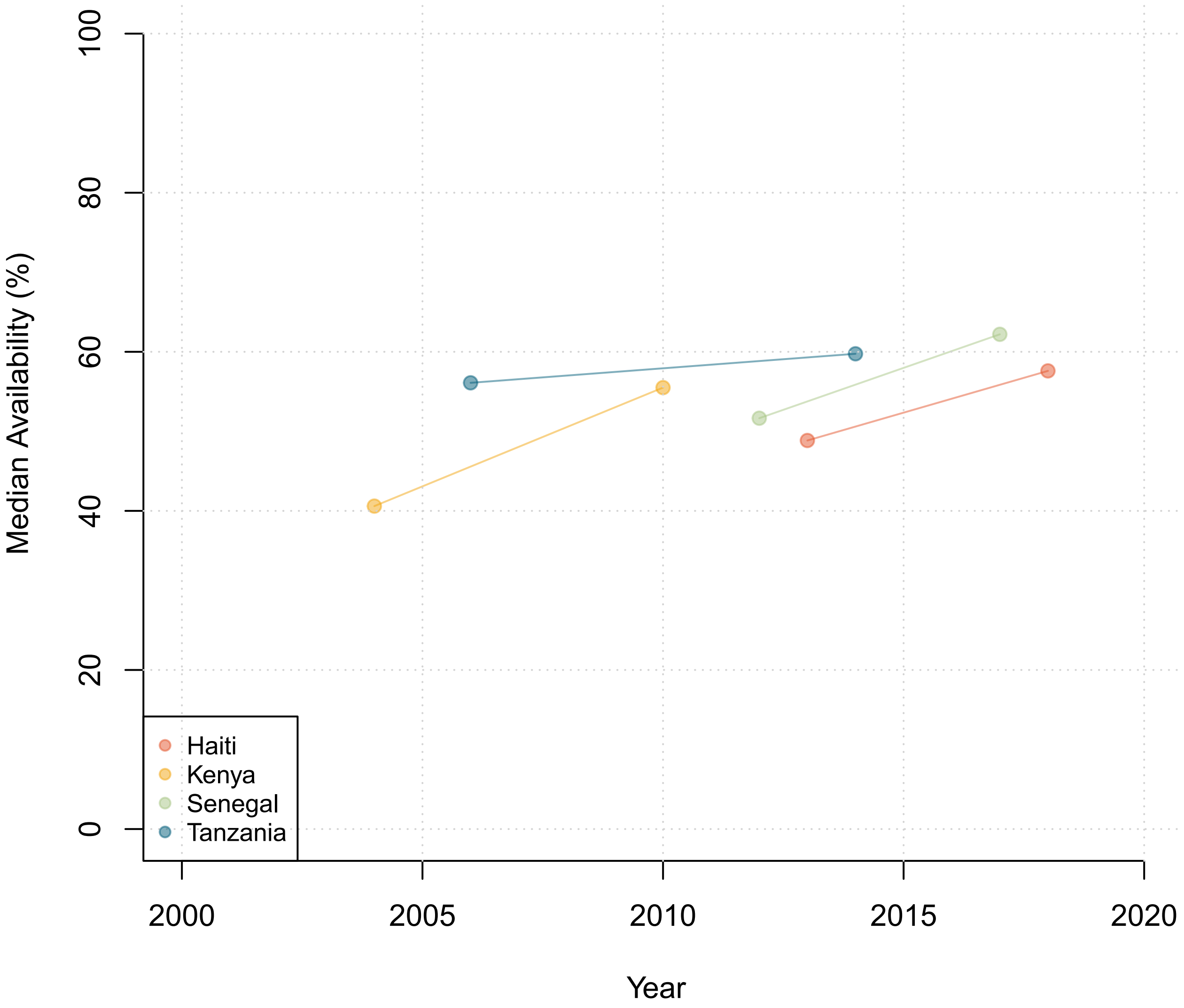

Figure S3. Plot over time of median availability for each survey in countries that underwent two surveys. There is no apparent dependence on time (e.g., more change recently than in more remote years) other than roughly similar increas-es over time in each country.

Table S1. Diagnostic tests included within test groups

TEST NAME	TESTS INCLUDED - RECODE Version 1*	TESTS INCLUDED – RECODE Versions 5 and Higher
**HIV**	ELISA, dyna bead test, rapid HIV test, western blot, ELISA test sendout, western blot sendout, HIV PCR, HIV PCR sendout	ELISA, dyna bead test, rapid HIV test, western blot, ELISA test sendout, western blot sendout, rapid HIV at ANCs, rapid HIV test sendout, rapid HIV test at counseling sites, HIV PCR, HIV PCR sendout
**Malaria**	Microscopy, rapid antigen test, sendout tests	Microscopy, rapid antigen test, sendout tests
**Urine Protein**	Urine strip, acetic acid w/flame test for albumin, rrinalysis sendout	Urine strip, acetic acid w/flame test for albumin, urinalysis sendout, urine protein at ANC sites
**Urine Glucose**	Urine strip, benedict solution w/stove test, urinalysis sendout	Urine strip, benedict solution w/stove test, urinalysis sendout, urine glucose at ANC sites
**Urine Pregnancy**	Urine pregnancy test qualitative	Urine pregnancy test qualitative
**Syphilis**	RPR card test, VDRL test, VDRL w/ rotator	RPR card test, VDRL test, VDRL w/ rotator, TPHA test, rapid test, rapid test at ANC sites
**Glucometer**	N/A	Glucose meter with strips
**Microscopy**	Microscopes, slides	Microscopes, slides
**Hemoglobin**	Hematology analyzer, hemoglobinometer, litmus test, hematocrit, calorimeter, other tests	Hematology analyzer, hemoglobinometer, litmus test, hematocrit, calorimeter, hemocue, hematocytometer, other tests
**TB**	Rapid TB PCR, TB culture, acid fast bacilli smear	Rapid TB PCR, TB culture, acid fast bacilli smear by LED or bright-field microscopy
**Chemistry Analyzer**	Chemistry analyzer, blood chemistry sendout	Chemistry analyzer, blood chemistry sendout
**Heme Analyzer**	Hematology analyzer	Hematology analyzer
**Gram stain**	Gram stain, gram stain sendout	Gram stain, gram stain sendout
**X-ray**	X-ray	Digital X-ray, analog X-ray
**Ultrasound**	Ultrasound	Ultrasound
**CT**	Computerized tomography	Computerized tomography

* SPA surveys have evolved over time, with slightly different questions and associated recoding. Recoding facilitates comparison between years and countries.

Table S2: Facilities by country included in each tier.

Country	Year	Merged Tier	Facilities included in SPA	Description
**Bangladesh**	2014	Basic Primary	Community clinic, NGO Clinic	community level health care provided by domiciliary health providers
		Advanced Primary	Upazila Health complex, Maternal and Child welfare center, Union Health and family Welfare Center, Union Subcenter, Rural Dispensary,	Upazila health complex consists of primary care services and the upazila hospital which has 30-50 beds.
		Hospital	District Hospital, Private hospital (with > 20 beds), NGO Hospital	Include Medical Institutes, Specialized Healthcare centers, Medical college hospitals, and Infectious disease hospitals
**Haiti**	2013	Basic Primary	Health Center without beds, Dispensary	
		Advanced Primary	Health Center with beds	
		Hospital	University Hospital, Regional Hospital, Community referral Hospital, Hospital	Provide diagnostic, radiological, emergency, surgical, obstetrics and pediatrics services
	2018	Basic Primary	Health Center without beds, Dispensary, Community Health center	Community Health centers sit at the neighborhood or district Level, Health centers are located at the commune level.
		Advanced Primary	Health Center with beds	
		Hospital	University Hospital, Regional Hospital, Community referral Hospital, Other Hospital	Provide diagnostic, radiological, emergency, surgical, obstetrics and pediatrics services
**Kenya**	2004	Basic Primary	Clinic, Dispensary, Maternity, Stand-alone (VCT, PMTCT, ART)	VCT: Volunteer Counseling and testing for HIV; PMTCT: Prevention of maternal to child transmission of HIV; ART: Anti-retroviral therapy
		Advanced Primary	Health Center	

		Hospital	Hospital	Includes National Referral Facilities
	2010	Basic Primary	Clinic, Dispensary, Maternity, Stand-alone (VCT)	
		Advanced Primary	Health Center	
		Hospital	National referral hospital, Provincial Hospital, District Hospital, Sub-district Hospital, Other Hospital	
**Malawi**	2014	Basic Primary	Clinic, Dispensary, Maternity, Health post	
		Advanced Primary	Health Center	
		Hospital	Central Hospital, District Hospital, Rural/Community Hospital, Other Hospital	Rural and community Hospitals act as referral facilities for district hospitals while also providing Curative services in their region. Central Hospitals offer professional training, conduct research and provide support to districts.
**Namibia**	2009	Basic Primary	Clinic, Free standing (VCT), Sick bay	
		Advanced Primary	Health Center	
		Hospital	Hospital	
**Nepal**	2015	Basic Primary	Health Post, Sub-Health post, Stand alone (HTC)	HTC: HIV Testing and Counseling sites
		Advanced Primary	Primary Health care center, Urban Health Center	
		Hospital	Central Govt. Hospital, Regional Govt. Hospital, Sub-regional Govt Hospital, Zonal govt. Hospital, District Govt. Hospital, Other Non - state owned Hospital	Provide Inpatient, outpatient, emergency, obstetric and neonatal care services. Central Hospitals also offer professional training and conduct research while providing support to districts
**Rwanda**	2007	Basic Primary	Dispensary, Health post, Clinic	

		Advanced Primary	Health Center, Polyclinic	
		Hospital	Hospital	
**Senegal**	2012	Basic Primary	Clinic	
		Advanced Primary	Health Center	
		Hospital	Hospital	
	2017	Basic Primary	Clinic	
		Advanced Primary	Health Center	
		Hospital	Hospital	
**Tanzania**	2006	Basic Primary	Dispensary, Stand-alone	Provide preventive care and promote healthy practices
		Advanced Primary	Health Center	Provide preventive and curative services
		Hospital	Hospital	
	2014	Basic Primary	Clinic, dispensary	Provide preventive care and promote healthy practices
		Advanced Primary	Health Center	
		Hospital	National Referral hospital, Regional Hospital, District Hospital, District - designated Hospital, Other hospital (Private)	Undertake curative and rehabilitative services, limited amount of preventive care and health promotion.
**Uganda**	2007	Basic Primary	Health Center II	Parish level with a catchment population of 5,000
		Advanced Primary	Health center IV, Health center III	Health Center III is the sub-county Level with a catchment population of 20,000; Health Center IV is the county Level with a catchment of 100,000
		Hospital	Hospital	Include National-level institutions, National and regional referral Hospitals, District Health services. Hospitals have a catchment population of 500,000 - 2,000,000

*Where available in SPA reports or government websites.

Table S3: Diagnostic availability for 16 different tests (%), with unweighted averaging across all countries, by tier.

RANK	Basic Primary		Advanced Primary		Hospital	
1	Malaria	37.1	Malaria	76.7	Malaria	91.6
2	HIV	32.9	HIV	72.2	HIV	88.6
3	Urine Pregnancy	32.2	Urine Protein	60.1	Urine Protein	86.2
4	Urine Protein	29.8	Urine Glucose	57.3	Urine Glucose	82.9
5	Urine Glucose	27.1	Urine Pregnancy	56.6	Syphilis	81.3
6	Glucometer	19.9	Syphilis	55.3	Urine Pregnancy	80.6
7	Syphilis	15.8	Microscopy	47.3	Microscopy	75.7
8	Hemoglobin	14.3	Hemoglobin	33	Chemistry Analyzer	62.3
9	Microscopy	12.7	Glucometer	32.5	X-ray	61.5
10	Ultrasound	1.2	Ultrasound	3.1	TB	58.8
11	TB	N/A	TB	N/A	Heme Analyzer	51.4
12	Chemistry Analyzer[1]	N/A	Chemistry Analyzer	N/A	Ultrasound	50.7
13	Heme Analyzer[2]	N/A	Heme Analyzer	N/A	Gram Stain	45.8
14	Gram Stain	N/A	Gram Stain	N/A	CT	6.1
15	X-ray	N/A	X-ray	N/A	Hemoglobin	N/A
16	CT	N/A	CT	N/A	Glucometer	N/A

[1]Chemistry Analyzer calculates the concentration of electrolytes, glucose, renal function, and liver function. [2]Heme Analyzer measures complete blood count: includes red blood cell (RBC), white blood cell (WBC), hemoglobin, platelet counts, and hematocrit levels.

Table S4: Ranking of countries (and year of survey) based on median availability of diagnostic testing (%) in each tier.

RANK	Basic Primary		Advanced Primary		Hospital	
1	Namibia, 2009	86.2	Namibia, 2009	97.9	Namibia, 2009	86.7
2	Senegal, 2017	68.5	Senegal, 2017	72.2	Kenya, 2010	84.3
3	Haiti, 2018	34.0	Kenya, 2010	68.9	Nepal, 2015	80.0
4	Kenya, 2010	32.9	Haiti, 2018	64.5	Uganda, 2007	79.6
5	Rwanda, 2007	25.0	Tanzania, 2014	62.2	Haiti, 2018	71.3
6	Tanzania, 2014	23.6	Rwanda, 2007	47.9	Rwanda, 2007	70.2
7	Malawi, 2014	14.7	Uganda, 2007	34.1	Tanzania, 2014	70.2
8	Uganda, 2007	8.0	Nepal, 2015	33.6	Bangladesh, 2014	62.1
9	Bangladesh, 2014	5.0	Malawi, 2014	9.2	Malawi, 2014	60.7
10	Nepal, 2015	3.3	Bangladesh, 2014	5.7	Senegal, 2017	60.2

Table S5. Regression results of country median availability of diagnostics vs log10(income) per capita with covariates.

Coefficient	Description	Estimate	Confidence Interval	Probability
Log10(Income)	Income per capita Log	12.2	-27.1 to 51.6	0.5271
Year+	Year of survey	-1.1	-2.9 to 0.7	0.2124
Advanced Primary	Health Tier	-182.5	-346.9 to -18.2	0.0311
Basic Primary	Health Tier	-211.6	-376.0 to -47.2	0.0139
IncomeLog:Advanced Primary	Interaction of Income and Tier	54.2	-1.3 to 109.7	0.0553
IncomeLog:Basic Primary	Interaction of Income and Tier	57.4	1.9 to 112.9	0.0432

Overall regression (F-statistic: 9.242 on 6 and 23 DF, p-value: 3.352e-05, Multiple R-squared: 0.7068, Adjusted R-squared: 0.6304)

+ Year refers to the year the survey was conducted in the specific country, to distinguish the survey included in the analysis when more than one survey was conducted in that country.

Table S6. Regions identified as outliers compared to other within-country regions

Outlier	Tier	Country	Region	Population Density	Population Density 25th percentile*	Population Density 50th percentile	Population Density 75th percentile	Availability (%)
High	Primary	Nepal	Far-western terai	288.2	134.6	180.4	451.2	21.4
High	Primary	Tanzania	Dar es Salaam	3133.0	32.2	54.0	117.2	53.8
High	Primary	Tanzania	Mjini Magharib	2581.0	32.2	54.0	117.2	50.4
High	Primary	Uganda	Kampala	6606.3	128.9	150.0	275.0	62.5
High	Secondary	Malawi	South	243.9	147.7	211.0	227.5	13.0
High	Secondary	Tanzania	Kusini Unguja	135.3	32.2	54.0	117.2	100.0
High	Secondary	Uganda	Kampala	6606.3	128.9	150.0	275.0	83.2
Low	Secondary	Kenya	Nairobi	4457.9	39.5	194.0	454.4	33.3
Low	Secondary	Kenya	Northeastern	18.1	39.5	194.0	454.4	22.2
Low	Tertiary	Kenya	Northeastern	18.1	39.5	194.0	454.4	56.8
Low	Tertiary	Namibia	Erongo	2.3	0.8	4.6	9.2	66.7
Low	Tertiary	Namibia	Kavango	4.6	0.8	4.6	9.2	75.0
Low	Tertiary	Namibia	Khomas	9.3	0.8	4.6	9.2	90.0
Low	Tertiary	Namibia	Otjozondjupa	1.4	0.8	4.6	9.2	85.7
Low	Tertiary	Senegal	Dakar	6823.2	55.3	75.3	215.7	38.7
Low	Tertiary	Senegal	Kolda	57.8	55.3	75.3	215.7	0.0
Low	Tertiary	Senegal	Thies	315.7	55.3	75.3	215.7	76.7
Low	Tertiary	Tanzania	Kusini Unguja	135.3	32.0	50.4	97.3	0.0
Low	Tertiary	Tanzania	Mjini Magharib	2581.0	32.0	50.4	97.3	50.0
Low	Tertiary	Tanzania	Tanga	30.0	32.0	50.4	97.3	26.4

*Percentiles with respect to regional population densities within-country

Table S7. Regression results of region availability of diagnostics vs population density with covariates.

Coefficient	Description	Estimate	Confidence Interval	Probability
Population Density (population/area)	Scaled to mean and standard deviation, within country	-1.8	-5.1 to 1.6	0.2994
Haiti, 2018	Survey, year^{+}	39.2	29.3 to 49.1	9.05e-14
Kenya, 2010	Survey, year	35.0	24.6 to 45.4	1.47e-10
Malawi, 2014	Survey, year	4.0	-9.9 to 17.8	0.5717
Namibia, 2009	Survey, year	71.3	61.9 to 80.7	<2e-16
Nepal, 2015	Survey, year	17.1	7.7 to 26.5	0.0004
Rwanda, 2007	Survey, year	24.0	12.3 to 35.8	7.3e-05
Senegal, 2017	Survey, year	56.0	46.6 to 65.4	<2e-16
Tanzania, 2014	Survey, year	28.8	20.4 to 37.2	9.32e-05
Uganda, 2007	Survey, year	20.4	10.2 to 30.5	9.32e-05
Advanced Primary	Health tier	-19.2	-23.9 to -14.6	1.1e-14
Basic Primary	Health tier	-45.8	-50.5 to -41.1	<2e-16
Population density:Advanced Primary	Interaction of population density and tier	1.98	-2.8 to 6.7	0.4112
Population Density:Basic Primary	Interaction of population density and tier	5.4	0.7 to 10.1	0.0252

Overall regression (F-statistic: 56.23 on 14 and 319 DF, p-value: < 2.2e-16, Multiple R-squared: 0.7116, Adjusted R-squared: 0.699)

Since the regional population density is z-score transformed within-country, the regression coefficient of 5.4 for the interaction term Population Density:Basic Primary means a population density shift of 1 (~34% shift from the mean population density) is associated with an increase in availability at the Basic Primary tier of 5.4% (e.g., shift from 50% to 55.4%).

Separate linear regressions performed independently for each tier, with Country as covariate, produced statistically significant coefficiencts for population density at Primary Basic (p=0.00149) but not Primary Advanced (p=0.881) or Hospital (p=0.297573) tiers.
